# Root Canal Instrumentation: Current Trends and Future Perspectives

**DOI:** 10.7759/cureus.58045

**Published:** 2024-04-11

**Authors:** Swati Srivastava

**Affiliations:** 1 Department of Conservative Dental Sciences, College of Dentistry, Qassim University, Buraidah, SAU

**Keywords:** advancements, rotary nickel-titanium, filing system, current trends, instrumentation techniques, root canal therapy

## Abstract

The evolution of root canal instrumentation techniques has significantly impacted the field of endodontics, enhancing both the efficiency and outcomes of treatments. This review outlines the transition from manual to mechanical and rotary instruments, highlighting the role of nickel-titanium (NiTi) alloys and smart technologies in advancing procedural precision and reducing patient discomfort. Key historical developments and technological innovations, such as digital imaging and navigation systems, are explored for their contributions to improved clinical outcomes and patient satisfaction. Additionally, the review addresses the challenges presented by the complex anatomy of the root canal system and the advent of current instrumentation techniques. The potential of emerging trends, including artificial intelligence and advances in materials science, is discussed in the context of future endodontic practices. Despite the progress, challenges related to using advanced instrumentation methods, ethical considerations, and the cost factor of new technologies persist. The present review underscores the ongoing need for research and development to further refine root canal instrumentation techniques, ensuring that advancements in endodontic care remain patient-centered and accessible.

## Introduction and background

Root canal treatment is a cornerstone in preserving dental health, critical for the retention of teeth compromised by infection or necrosis [1]. Recent advancements in root canal instrumentation have significantly augmented procedural efficacy and patient outcomes. Historically, manual instrumentation was the standard, but with the advent of mechanical and rotary instruments, the precision and speed of root canal treatments have improved dramatically [2].

Schilder (1974) transformed endodontic philosophy by introducing the principles of three-dimensional obturation, highlighting the significance of thorough canal shaping [3]. However, while Schilder's principles were groundbreaking, the primary objective of root canal preparation extends beyond his aims. It aims to prevent and facilitate the healing of apical periodontitis, making clinical outcomes the principal criterion for evidence-based assessment. Recent advancements, such as the development of single-file systems, have furthered procedural efficiency while maintaining treatment efficacy [4]. With the advent of nickel-titanium (NiTi) rotary files, a new era of flexibility and efficiency in canal instrumentation has helped reduce the incidence of file breakage and procedural errors [5]. Moreover, with the use of engine kinematics having reciprocating motion of rotary files, decreased chances of microcrack propagation and vertical root fracture are observed [6].

More recently, advancements in digital imaging and navigation technologies have begun to influence endodontic instrumentation techniques, promising even greater precision and safety [7]. These studies underscore a trend toward minimizing patient discomfort while maximizing the procedural success rate. This review aims to dissect the evolution of root canal instrumentation techniques from manual to mechanical, highlighting key studies and innovations. It will explore the impact of these advancements on clinical outcomes, patient satisfaction, and procedural efficiency, setting the stage for a discussion on the future directions of endodontic instrumentation.

Methodology

**Figure 1:**
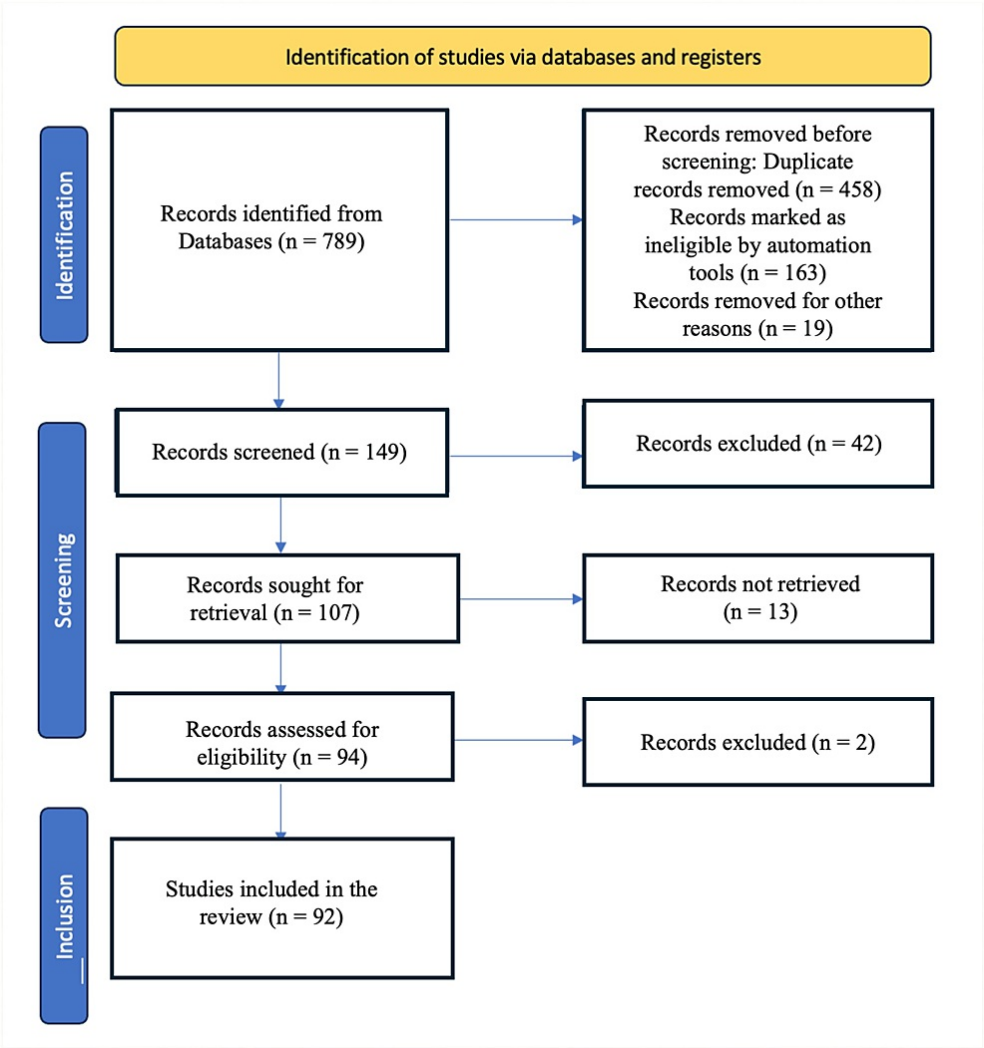
Flowchart showing the step-by-step identification of the studies via databases

To conduct a literature survey on 'Root Canal Instrumentation: Current Trends and Future Perspectives,' a search was conducted in January 2024 across various electronic databases, including PubMed, Scopus, EMBASE (Excerpta Medica Database), Cochrane Library, and Science Direct. The search utilized MeSH terms/keywords such as "Root Canal," "Instrumentation," and "Current Trends.” In addition to the electronic search, cross-references and textbooks were manually searched for relevant articles. The inclusion criteria included articles published in the English language from January 2010 to January 2024 that fulfilled the study objectives. The article selection process involved assessing the inclusion and exclusion criteria, as well as conducting a quality assessment. Out of the initial 789 articles identified, 124 were selected based on their titles and abstracts. After evaluating the full texts and applying the inclusion and exclusion criteria, 92 articles were chosen for the review, meeting the study's criteria (Figure 1). Animal-based studies and narrative reviews on “Root Canal Instrumentation” were excluded from the selection process.

## Review

Historical perspective

The historical perspective on root canal treatment reflects a remarkable journey of innovation and advancement. Initially, root canal procedures were rudimentary, focusing merely on alleviating pain without necessarily preserving the tooth. As dental science progressed, so did the understanding of endodontic principles, leading to the development of manual instrumentation techniques aimed at cleaning and shaping the root canal system more effectively [8].

The evolution from manual to mechanical instrumentation marked a significant turning point. Early instruments were simple files made from stainless steel, designed for hand use, which made the procedure time-consuming and technique-sensitive [9]. The introduction of NiTi alloys revolutionized this process, offering greater flexibility and resistance to fatigue, enabling the development of rotary and reciprocating systems that enhanced the efficiency, accuracy, and predictability of root canal treatments (Table 1) [10]. These technological advancements, coupled with an improved understanding of root canal anatomy and the microbial etiology of endodontic infections, have fundamentally changed the approach to root canal therapy. The shift toward mechanical instrumentation has not only improved the quality of treatment but also significantly reduced the physical strain on practitioners and discomfort for patients, marking a new era in endodontic therapy [5].

**Table 1:** Evolution of root canal instrumentation, from historical to modern techniques NiTi: nickel-titanium

Author/manufacturer (year)	Objective/invention	Findings/development
William H Rollins (1889) [11 ]	Development of the first endodontic handpiece.	Use of specially designed needles, which were mounted into a dental handpiece with a 360-degree rotation. To avoid instrument fractures, rotational speed was limited to 100 rpm.
Oltramare (1892) [12]	The first description of the use of rotary devices.	Reported the use of fine needles with a rectangular cross-section, which could be mounted into a dental handpiece. These needles were passively introduced into the root canal to the apical foramen and then the rotation started.
Austrian company W&H (1928) [13]	Cursor filing contra-angle handpiece.	This handpiece created a combined rotational and vertical motion of the file.
Austrian company W&H (1958) [13]	The Racer handpiece.	Worked with a vertical motion.
MicroMega, Besancon, France (1964) [13]	The Giromatic handpiece.	Worked with a reciprocal 90-degree rotation.
Levy (1984) [14]	The Canal Finder System.	The Canal Finder was the first endodontic handpiece with a partially flexible motion. It was an attempt to make the root canal anatomy or the root canal diameter the main influencing factor on the behavior of the instrument inside the canal.
Hulsmann (1997) [15]	Enlargement of root canals.	Root canals should be enlarged with three- or four-sided broach, tapering to a sharp point. This instrument is employed to enlarge the canal and give it a regular shape.
Bergmans et al. (2001) [16]	To review current endodontic instruments and shaping techniques.	Introduction of NiTi alloys and the impact on root canal instrumentation efficiency.
Spili et al. (2005) [17]	Description of the evolution of rotary files and their influence on treatment success rates.	With appropriate manipulation and special attention to the equipment used, NiTi systems are safe with a minimal incidence of instrument failure.
Metzger et al. (2010) [18]	Introduction of a new concept, the self-adjusting file (SAF), and discuss its unique features compared with current rotary NiTi file systems.	A three-dimensional adaptation to the shape of the root canal, including adaptation to its cross-section. One file is used throughout the procedure, during which it changes from an initially compressed form to larger dimensions. Canal straightening and canal transportation of curved canals are largely avoided because of the lack of a rigid metal core. core. The high mechanical durability and hollow design of the SAF allow for continuous irrigation during instrumentation.
Kuttler et al. (2012) [19]	Impact of instrument fracture on the outcome of endodontic treatment.	In the hands of experienced operators, endodontic instrument fracture, in particular rotary NiTi, had no adverse influence on the outcome of nonsurgical root canal treatment and retreatment.
Peralta-Mamani et al. (2019) [20]	To systemically review in-vitro studies comparing the efficiency of manual and rotary instrumentation in permanent teeth.	The rotary instrumentation presents better results regarding root canal transportation, the ability of centralization, and the shaping of canals. Manual instruments are safer and produce less smear layer and dentinal debris, have more instrumented canal surface, and cause fewer dentinal defects.

Anatomy of the root canal system

The complexity of the root canal system, with its intricate anatomy and variation, poses significant challenges in endodontic treatments. The root canal anatomy is not uniform; variations such as the number of canals, their shapes, sizes, and curvatures, as well as the presence of accessory canals and anatomical anomalies like isthmuses, significantly influence the approach to root canal instrumentation [21]. For instance, molars, with their multiple canals and potential for complex configurations, require detailed attention to ensure thorough cleaning and shaping [22]. The variability among individual teeth and even within the same tooth complicates the instrumentation process, necessitating advanced techniques and tools to adequately address these anatomical challenges and achieve successful endodontic outcomes (Table 2).

**Table 2:** Challenges in endodontic instrumentation across different tooth anatomies

Tooth type	Anatomical challenge	Instrumentation challenge
Molars	Complex root canal systems with multiple canals and branches [22].	Accessing and cleaning all canals thoroughly, avoiding perforation, and managing curved or narrow canals [22].
Premolars	Variable number of canals and root configurations. Some have a single canal, while others may have two or more [21].	Identifying and treating all present canals, especially when they are small or have unusual configurations [21].
Canines	Long roots with a single canal that may have curvature [22].	Navigating the length and curvature without causing instrument fracture or canal transportation [22].
Incisors	Generally, a single straight canal, but lateral incisors may have curvature or extra canals [22].	Straightforward in most cases, but detecting and managing extra canals or curvatures in lateral incisors can be challenging [22].
Maxillary molars	Often have three roots with at least three canals, but the presence of a fourth canal (MB2) in the mesio buccal root is common [22].	Locating and adequately cleaning the MB2 canal, which can be difficult to detect and access [22].
Mandibular molars	Typically have two roots, but the configuration of the canals, especially in the distal root, can vary [22].	Managing variations in canal configuration, especially the presence of a middle mesial canal or retreating previously untreated anatomy [22].

Current trends in root canal instrumentation

Root canal treatment has seen significant advancements in techniques and technologies over the years, aiming to improve the efficacy, efficiency, and outcomes of endodontic procedures. The primary goal of root canal instrumentation is the removal of infected tissue, debris, and bacteria from the root canal system, thereby preparing the canal for obturation. Standard practices have evolved from manual filing techniques to sophisticated mechanical instrumentation supported by advanced imaging and irrigation methods [23]. The emphasis is on thorough canal cleaning while preserving tooth structure, minimizing treatment time, and enhancing patient comfort.

Rotary NiTi Instruments

Rotary NiTi instruments have revolutionized root canal preparation by offering greater flexibility, efficiency, and safety compared to traditional stainless-steel files [24]. The mechanical characteristics of NiTi instruments are impacted by various factors including cross-section, flute design, raw material, and manufacturing methods. By incorporating surface engineering techniques such as implantation or electropolishing, as well as microstructure control methods like heat treatment or innovative manufacturing techniques, into the design of endodontic files, more desirable outcomes have been achieved in terms of instrument flexibility, fatigue resistance, and cutting efficiency [25].

Heat treatment, also known as thermal processing, stands as one of the fundamental methods for adjusting the transition temperatures of NiTi alloys and influencing the fatigue resistance of NiTi endodontic files. This has led to the development of the next-generation endodontic instruments. M-Wire (Dentsply Tulsa Dental Specialties, Tulsa, Oklahoma, United States) instruments are made of NiTi wire blanks that contain the substantially stable martensite phase under clinical conditions. Some of the available systems are Dentsply's ProFile GT Series X, ProFile Vortex, ProTaper Next, Path Files, WaveOne, and Reciproc (Dentsply Sirona, Charlotte, North Carolina, United States) [26]. The literature indicates a notably enhanced cyclic fatigue resistance of M-wire, as observed in instruments like ProTaper Next (Dentsply Sirona, Charlotte, North Carolina, United States), compared to conventional NiTi instruments. However, several studies have reported no significant difference in cyclic fatigue between M-wire and conventional NiTi files [27,28].

Another type of heat treatment of conventional NiTi wires in the austenite phase can result in the transformation into an intermediate rhombohedral crystal structure known as the R-phase, situated between the austenite and martensite phases. The R-phase exhibits favorable superelasticity and shape memory effects. Consequently, an instrument crafted from R-phase wire possesses greater flexibility. Twisted files are an example of the R-phase [29]. The literature indicates that the R-phase instruments typically exhibit lower torsional strength compared to conventional NiTi files [30,31].

CM-Wires (DS Dental, Johnson City, Tennessee, United States) are controlled memory NiTi alloys. They represent a novel NiTi alloy renowned for its flexible properties, manufactured through a proprietary thermo-mechanical process. This unique composition enables instruments to be pre-curved before placement into the root canal, with sterilization restoring them to their original shape. Systems using CM-Wire technology are Hyflex CM (Coltene Holding AG, Altstätten, Switzerland), Typhoon CM (Clinician's Choice Dental Products, Inc., New Milford, Connecticut, United States), and ProFile Vortex Blue (Dentsply Sirona, Charlotte, North Carolina, United States). However, they possess a significant drawback of an increased susceptibility to permanent plastic deformation during use. Because of this weakness, it is recommended that these instruments be used only once [32].

Max-Wire (martensite-austenite-electropolish-fileX), recently introduced by FKG Dentaire (Le Locle, Neuchatel, Switzerland), represents the first endodontic file system to combine both shape memory effect and superelasticity in a single system for clinical applications. The two available marketed instruments of Max-Wire are XP-endo Shaper and XP-endo Finisher, both developed by FKG Dentaire. These files are initially relatively straight in their martensitic phase at room temperature. However, when exposed to intracanal temperature, they undergo a phase transformation to the austenitic phase, causing them to adopt a curved shape. As a result, they exhibit the shape memory effect when inserted into the canal (transitioning from the martensite state to the austenite state) and demonstrate superelasticity during canal preparation. The curved shape of these files allows them to adapt exceptionally well to canal irregularities [33].

Studies have reported a significantly increased cyclic fatigue resistance of XP-endo Shaper compared to other systems such as Hyflex CM, Vortex Blue, and iRace (FKG Dentaire, Le Locle, Neuchatel, Switzerland) [34,35].

Reciprocating Single-file Systems

The primary cause of instrument fracture still stems from continuous rotational movement. To effectively mitigate the risk of torsional fracture, an alternative approach involving an alternating clockwise and counter-clockwise movement with varying angles, known as reciprocating movement, has been reintroduced in recent years [36]. Modern reciprocation involves a combined movement that includes a cutting phase in one direction of rotation, immediately followed by a phase where the instrument engages in the opposite rotational movement. This type of motion effectively decreases the risk of torsional fracture, with reciprocating angles varying depending on the instrument. Typically, the first cutting angle is larger than the second one, creating a rotational effect that facilitates the instruments' advancement inside the root canal [37]. WaveOne and Reciproc are based on reciprocating motion. In addition to decreasing the risk of torsional fracture, reciprocating movement has demonstrated increased resistance to cyclic fatigue fracture for all instruments. This motion decreases the likelihood of file separation [23]. Reciprocating systems are praised for their simplicity and time efficiency, as a single file is often sufficient to prepare the canal. Nonetheless, the potential for inadequate canal shaping exists, especially in highly curved or complex canals.

Adjunctive Technologies

Ultrasonics: Ultrasonic instruments are used for various purposes, including irrigation, removal of canal obstructions (e.g., broken instruments and calcifications), and endodontic microsurgery [38]. Ultrasonics enhance the cleaning efficacy by agitating the irrigation solutions, improving their penetration and the removal of debris from the canal system [39].

Microscopy and magnification: The use of dental operating microscopes and magnification devices has become a standard in modern endodontics [40]. These tools greatly enhance the visualization of the canal system, enabling the identification and treatment of complex anatomical variations, additional canals, and canal pathologies that would be challenging to manage with the naked eye [41].

Irrigation systems and solutions: Effective irrigation is crucial for the removal of debris, biofilm, and bacteria from the root canal system. Advanced irrigation systems, such as sonic and ultrasonic agitation, have been developed to improve the distribution and effectiveness of irrigation solutions. Commonly used solutions include sodium hypochlorite, ethylenediamine tetraacetic acid (EDTA), and chlorhexidine, each with specific roles in cleaning, disinfection, and the removal of the smear layer [42].

However, as the search for the ideal irrigant continues, combinations of two or more solutions have emerged to blend their desired properties. Notably, commonly used irrigants such as sodium hypochlorite (NaOCl), EDTA, and chlorhexidine (CHX) are occasionally mixed with surfactants to decrease their surface tension [43]. This practice is rooted in the misconception that lower surface tension can improve irrigant penetration into the root canal system [44, 45]. Some available mixtures, like BioPure MTAD (Dentsply Sirona, Charlotte, North Carolina, United States), Tetraclean (Ogna Laboratori Farmaceutici, Muggiò, Italy), and QMix (Dentsply Sirona, Charlotte, North Carolina, United States), incorporate an antimicrobial agent, a chelating agent, and one or more surfactants. These mixtures are often recommended for final rinsing instead of EDTA to eliminate the smear layer and enhance the antimicrobial effect of NaOCl [46-48].

Materials and tools

The field of endodontics has witnessed significant advancements in the materials and tools used for root canal instrumentation, largely driven by the quest for improved treatment outcomes and patient comfort. At the heart of these advancements are the development and refinement of NiTi alloys, which have revolutionized the design and function of endodontic instruments [49]. Understanding these aspects is crucial for clinicians to make informed decisions that enhance procedural success and patient satisfaction. The introduction of NiTi alloys and the evolution of tool designs have significantly impacted the practice of endodontics. NiTi-based rotary and reciprocating instruments offer a blend of flexibility, strength, and efficiency, allowing for safer and more effective canal preparation [50].

Furthermore, advancements have been made in improving the efficacy of irrigant activity through negative apical pressure systems, as well as sonic and ultrasonic agitation techniques [51]. Employing these modern techniques for cleaning and shaping the root canal system should instill greater confidence and predictability in managing endodontic disease (Table 3).

**Table 3:** Materials and tools in modern endodontic instrumentation NiTi: nickel-titanium

Author (year)	Material/tool	Characteristics	Advantages	Clinical implications
Duque et al. (2020) [52]	NiTi alloys	High flexibility and resistance to cyclic fatigue.	Reduced risk of instrument fracture and improved ability to navigate curved canals.	Enhances the safety and efficiency of root canal instrumentation, especially in complex anatomical situations.
Kuzekanani et al. (2018) [53]	Rotary NiTi instruments	Engine-driven and designed for efficient canal shaping.	Faster canal preparation with consistent results and minimizes manual effort.	The superelasticity and shape memory of these instruments minimize the risk of canal transportation, thereby saving time for both patients and clinicians.
Grande et al. (2007) [54]	Reciprocating NiTi systems	Single-file systems that use a reciprocating motion to clean and shape canals.	Reduces the risk of torsional fracture and increased resistance to cyclic fatigue.	Particularly useful in straight or slightly curved canals, but may require complementary instruments for complex cases [55-57].
Paixao et al. (2022) [58]	Ultrasonic instruments	Use ultrasonic vibration to assist in endodontic procedures, including irrigation and debris removal.	Enhanced cleaning and debridement capabilities with improved access to difficult areas.	Root canals with ultrasonically activated irrigants have a remarkedly high push-out bond strength value between root canal sealers and root canal dentin.
Tomson et al. (2016) [59]	Irrigation systems	Advanced devices designed to optimize the delivery and agitation of irrigation solutions.	Improves the effectiveness of irrigation solutions in removing debris and disinfecting canals.	Critical for achieving optimal disinfection of the root canal system, though the choice of system can impact procedure time and cost.

Clinical outcomes and complications

The evolution of instrumentation techniques in endodontics, particularly with the introduction of NiTi rotary and reciprocating instruments, has significantly improved the success rates of root canal treatments. Studies have reported success rates ranging from 85% to 97% for root canal treatments using these modern techniques [54]. The use of NiTi instruments, along with advanced imaging techniques like cone beam CT (CBCT) and enhanced irrigation protocols, has improved the thorough cleaning and shaping of root canals, effectively reducing the bacterial load and increasing the likelihood of treatment success [58]. While modern instrumentation techniques have improved the success rates of endodontic treatments, they are not devoid of potential complications. Some of the common complications are given below.

Instrument Fracture

NiTi instruments, despite their flexibility and strength, can fracture within the canal if used beyond their fatigue limit or if the canal anatomy is highly complex. When this occurs, attempts may be made to remove the fractured segment using ultrasonics or micro-tweezers, although sometimes it may need to be bypassed and encapsulated within the obturation material [60].

Ledging, Zipping, and Transportation

Incorrect use of instruments can lead to canal alterations, such as ledging (formation of a ledge in the canal wall), zipping (elongation of the apical foramen), and transportation (alteration of the canal's original path) [59]. To prevent or address these issues, clinicians might need to use smaller, more flexible files or adopt different instrumentation techniques to correct or minimize the deviation. The initial negotiation and bypassing of the ledge can be accomplished using a small file with a distinctive curve at the tip. Additionally, a gentle rotating motion of the file, coupled with a "picking" motion, can often facilitate the advancement of the instrument [61].

Postoperative Pain and Flare-ups

These are not uncommon and can result from various factors, including over-instrumentation, extrusion of debris or irrigation solution beyond the apical foramen, or complex microbial ecology within the canal [62]. Hence, flare-ups in endodontics are multifactorial, necessitating the consideration of preventive strategies [63]. Maintaining aseptic conditions is paramount to prevent flare-ups. Utilizing rotary-driven NiTi instruments with the crown-down technique, along with effective irrigation, has been shown to decrease flare-up incidences [64]. Additionally, employing devices for enhanced delivery of irrigants can further aid in preventing flare-ups. The use of intracanal medicaments between appointments and opting for single-visit treatment also holds the potential for reducing flare-up occurrences [65].

Perforations

Perforation of the canal wall can occur during instrumentation, especially in teeth with complex root anatomy or in cases of severe canal calcification [66]. Perforations are managed based on their location and size, with options including immediate sealing with biocompatible materials, such as mineral trioxide aggregate (MTA), to prevent bacterial contamination and promote healing [67].

Technological advancements

The field of endodontics has experienced a surge in technological innovations, significantly enhancing the precision, efficiency, and outcomes of root canal treatments [68]. These advancements span across various domains, including instrumentation techniques, digital dentistry, and materials science, each contributing to a more sophisticated and patient-friendly approach to endodontic therapy [5,25,69-70].

Recent Technological Innovations in Root Canal Instrumentation

In recent years, the development and refinement of instrumentation technologies have greatly improved the way root canals are prepared. Among these innovations are new generations of NiTi rotary instruments which have been developed with improved designs and alloy properties, offering greater flexibility, efficiency, and resistance to cyclic fatigue [71]. Significant changes include modifications in design, surface treatments, and thermal treatments. These changes aim to enhance the outcomes of root canal preparation and mitigate associated risks during root canal treatment. Heat treatment stands out as a fundamental approach to boosting the fatigue resistance and flexibility of NiTi endodontic instruments [72,73].

Furthermore, novel kinematics have been developed to provide enhanced safety and efficiency in root canal procedures. The torsional stresses experienced by off-centered instruments, in comparison to regular NiTi instruments with a centered axis of rotation, are more evenly distributed. This characteristic reduces the risk of fracture and minimizes the torque exerted by the instrument during cutting within the root canal [74].

The advantages of reciprocating movement have been rediscovered using NiTi instruments with increased taper. By applying the principles of modern reciprocating movements [75], it becomes feasible to cut with the tip of the instrument even in the absence of a glide path [76] and using a single instrument. This is attributed to the characteristics of the reciprocating movement, which reduce the risk of fracture when the instrument comes into contact with dentin, thus lowering the risk of fracture compared to constant rotation NiTi sequences [77].

Endodontic motors equipped with smart technology can adjust the speed, torque, and motion of the instrument in real time based on the resistance encountered, reducing the risk of instrument fracture [78]. The WaveOne motor is among the earliest tested motors and represents one of the initial technologies introduced to the market that provided reciprocating motion. The Jeni Motor's (Coltene Holding AG, Altstätten, Switzerland) angles are determined by the combination of rotation speed and duration in milliseconds, allowing adjustments in both clockwise and counter-clockwise directions. As a result, the Jeni Motor stands out as the only fully programmable motor capable of reciprocating movements [79].

The dynamic navigation system provides real-time guidance during the instrumentation process, allowing for precise navigation of instruments within the root canal system, and minimizing the risk of procedural errors [80]. The static navigation system involves a 3D endodontic guide, also known as EndoGuide, which serves as a template designed to direct drills to predetermined positions. It facilitates the location and exploration of root canal orifices, bone trephination, and root-end resection procedures [69].

The Role of Digital Dentistry

Digital dentistry has revolutionized endodontic diagnostics, planning, and treatment. CBCT imaging provides detailed 3D views of the tooth and surrounding structures, enabling accurate diagnosis of root canal anatomy, pathologies, and treatment complications [81]. Digital radiography offers instant images with lower radiation exposure, enhancing the efficiency and safety of endodontic treatments. 3D Printing technology is increasingly being used for the fabrication of custom dental guides, tools, and even synthetic teeth models for training and planning complex endodontic procedures.

Advances in Materials Science

Materials science has also seen significant developments, impacting the tools and substances used in root canal treatments. Bioceramic materials, such as mineral trioxide aggregate (MTA) and bioceramic sealers, have improved the sealing ability, biocompatibility, and healing outcomes of root canal fillings and repairs [82].

Improved irrigation solutions have led to the development of more effective antimicrobial agents and techniques for root canal disinfection [83]. Sodium hypochlorite, renowned for its potent organic tissue dissolution capabilities and broad-spectrum antibacterial properties, serves as an excellent disinfectant for various surfaces. Conversely, chelating agents like EDTA are employed to eliminate the inorganic components present in the smear layer. While this irrigation method effectively removes the smear layer, it may be less efficient in the apical third of the root canal [84]. Newer irrigants like QMix have been shown to have smear layer removal capability similar to MTAD (mixture of tetracycline isomer, acid, and detergent) but better than EDTA in the apical third area [85]. MTAD does not dissolve organic tissues, so it is best to use this after NaOCl at the end of the chemomechanical preparation step. MTAD is made up of three different compounds that together are expected to have a potent antibacterial action [84]. Tetraclean operates similarly to MTAD but has a reduced dosage of doxycycline and detergent. Despite this, Tetraclean exhibits substantial efficacy against both facultative and anaerobic bacteria. Studies have demonstrated that Tetraclean outperforms MTAD in terms of its effectiveness against planktonic and in-vitro biofilm Enterococcus faecalis cultures, as well as mixed-species biofilms [85]. The use of rotary brushes like Ruddle brush and canal brush helps efficiently clean the canal along with the irrigant [86]. The use of negative-pressure irrigation systems like RinsEndo (Duerr Dental Co, Bietigheim-Bissingen, Germany) and EndoVac (Discus Dental, Culver City, California, United States) has been shown to leave less debris behind than the traditional 30-gauge needle irrigation procedures [84].

Regenerative endodontics is exploring the use of growth factors, stem cells, and scaffolds to regenerate pulp tissue and promote the healing of periapical tissues. These technological advancements have collectively contributed to a paradigm shift in endodontic treatment, emphasizing minimally invasive techniques, enhanced patient comfort, and improved treatment outcomes. As research and development continue, the integration of these technologies into everyday clinical practice promises even greater advancements in the quality and efficiency of endodontic care [87].

Future perspective

The future of root canal instrumentation looks promising, with ongoing research focused on enhancing precision, efficiency, and patient outcomes. Emerging trends include the further development of instrumentation techniques that minimize tissue removal while maximizing cleaning effectiveness. Innovations such as more adaptable and intelligent file systems capable of adjusting to the unique anatomy of each canal are on the horizon. Furthermore, the integration of augmented reality (AR) in training and procedural guidance could revolutionize the way endodontists visualize and navigate the root canal system [88,89].

Artificial intelligence (AI) and machine learning are set to transform endodontics by improving diagnostic accuracy, treatment planning, and procedural outcomes. AI could be used to analyze radiographs and CBCT images more precisely, identifying anatomical features and pathologies that might be overlooked by the human eye [80]. Machine learning algorithms can also predict the most effective treatment approaches based on vast datasets, potentially customizing treatments to individual patient needs and anatomies. These technologies could also enhance the learning curve for dental professionals, offering personalized feedback and simulation-based training scenarios. The development of new materials, such as more resilient and flexible NiTi alloys and bioactive sealants, is expected to address current limitations in root canal instrumentation [80]. These materials can improve the longevity and success of endodontic treatments by enhancing the seal of the root canal system and promoting the healing of periapical tissues. Techniques that facilitate the regeneration of pulp tissue, leveraging advances in tissue engineering and regenerative medicine, hold the potential to not just treat but also restore the vitality of teeth previously deemed non-viable [90].

Challenges and considerations

Despite advancements, challenges remain, such as managing anatomical variations that complicate the instrumentation process [1]. Access to advanced technology is not uniform, with disparities influenced by geographical location and economic factors [91].

Additionally, maintaining the structural integrity of the tooth while ensuring thorough debridement poses a technical challenge that requires ongoing innovation in instrumentation techniques. The adoption of new technologies in endodontics raises ethical and cost considerations. There is a need to balance the benefits of advanced technologies with their accessibility to ensure equitable patient care. High costs associated with new instrumentation and materials may not be feasible for all practices or patients, potentially widening the gap in dental health disparities. Ethical considerations also include ensuring that the adoption of AI and other technologies does not compromise patient privacy or autonomy [92].

## Conclusions

The landscape of root canal instrumentation is evolving rapidly, driven by technological advancements and a deeper understanding of endodontic pathology and treatment. The future holds promise for even more precise, efficient, and patient-centered approaches to root canal therapy, with AI, machine learning, and new materials playing pivotal roles. However, the successful integration of these innovations will require careful consideration of clinical efficacy, ethical implications, and cost. Overcoming current challenges and limitations will necessitate a collaborative effort among researchers, clinicians, and industry stakeholders to ensure that the benefits of advanced endodontic care are accessible to all patients.
